# Protein–Carbohydrate Interactions in Food Matrices and Their Effects on Food Quality

**DOI:** 10.3390/foods15122213

**Published:** 2026-06-19

**Authors:** Muhammad Arif Ramzan, Anna Wang, Ligen Wu, Muhammad Abdul Haseeb

**Affiliations:** 1 School of Food Science and Technology, Henan University of Technology, Zhengzhou 450001, China; aafiramzan@gmail.com (M.A.R.); mabdulhaseeb326@gmail.com (M.A.H.); 2 National Engineering Research Center of Wheat and Corn Further Processing, Henan University of Technology, Zhengzhou 450001, China

**Keywords:** protein–polysaccharide complex, glycation, functional food, advanced glycation end products

## Abstract

The structure, functionality, nutritional value, and sensory properties of food are significantly influenced by interactions between proteins and carbohydrates. These interactions occur through hydrogen bonding, electrostatic forces, hydrophobic interactions, and, in many cases, the covalent attachment of sugars to proteins via the Maillard reaction. High starch content in food matrices promotes interactions between proteins and starch components such as amylose and amylopectin, affecting gelation, retrogradation, and thickening. These interactions improve shelf stability and product quality. Additionally, protein–carbohydrate interactions regulate nutrient digestibility and glycemic response, playing a crucial role in the development of functional foods for diabetes and weight management. In silico studies have demonstrated that dietary fibers like pectin and cellulose can improve water retention and textural properties in processed meat products. Furthermore, processing techniques such as enzymatic hydrolysis, fermentation, pulsed electric fields (PEF), and low-temperature drying have been found to improve the functional properties and shelf life of food products. This review synthesizes recent findings on protein–carbohydrate interactions and highlights their potential in creating healthier, more appealing, and sustainable foods that align with modern consumer preferences.

## 1. Introduction

Food quality is determined by nutritional value, structural stability, processing behavior, and sensory acceptability. Among the macromolecules that govern these properties, proteins and carbohydrates are the most abundant and the most interactive [1]. Their bonds (covalent or non-covalent) shape texture, digestion rate, mouthfeel, and consumer acceptance [2]. At the molecular scale, these interactions control gelation, emulsification, water-holding capacity, retrogradation, color, and flavor [3]. Non-covalent interactions include hydrogen bonding, electrostatic attraction, and van der Waals forces; covalent interactions are dominated by the Maillard reaction [4]. Recent bioinformatics and molecular docking studies have mapped how plant-derived polysaccharides such as pectin and cellulose bind to the principal meat proteins actin, myosin, and collagen. Hydrogen bonding and van der Waals forces between protein and polysaccharide are the major contributors to network strength in these systems and the resulting complexes increase firmness and water retention in fiber-enriched meat products [5].

The taste of roasted, baked, or grilled foods is shaped by flavor-active chemicals (pyrazines, aldehydes, furans) that are produced through the Maillard reaction between reducing sugars and amino acids [6]. Sweet lupine flour (SLF) is a good source of protein and polyphenols and has been used to make low-fat ice cream without skim milk powder.

The resulting formulation exhibited improved overrun, melting resistance, phenolic content, and antioxidant activity. It reduced the overall cost of making the products while the products were equally delicious [7].

Increasing attention has been directed toward the nutritional interactions between dietary proteins and carbohydrates [8]. When starch or fiber is combined, digestive enzymes require more time to hydrolyze starch, which leads to lower glycemic values and a steady release of energy, which is valuable for functional foods used to improve blood glucose regulation, satiety, and metabolic health. High-temperature and low-moisture processing conditions may reduce lysine bioavailability and promote the formation of advanced glycation end products (AGEs).

The qualities of taste, mouthfeel, and aroma are also noticeably influenced. Flavor-active chemicals (pyrazines, aldehydes, furans) are produced through the Maillard reaction, in which bonds are formed between reducing sugars and amino acids, shaping the taste of roasted, baked, or grilled foods. It has also been found that controlled glycation can lessen bitterness and give food more umami and sweetness [9]. Volatile compound release is slowed down by glycated matrices, giving people more time to enjoy the aroma as they chew [10]. Temperature, acidity, pressure, and fermentation and drying processes are all used by food scientists to manage chemical and physical reactions in food. An example is fermenting okara from soybeans, which helped the protein and fiber work well together, increased its antioxidant powers and made it easier to digest [11]. In the same way, using pulsed electric fields (PEF) and ultrasound glycation has demonstrated benefits in making complexes from proteins and carbohydrates perform better without many nutrient loss [12]. A trend in eco-friendly food design is to use products like citrus peel, oat fiber and tea polyphenols, which strengthen foods and can create complexes with their proteins [13]. As an illustration, combining phenolics with wheat protein and carbohydrates in baking gives dough a stable structure, and combining milk phenolics with tea strengthens the mixture’s stability and antioxidant activity [14]. In essence, these protein–carbohydrate interactions go beyond basic chemistry and are now used as strategies in the development of new foods.

Altering texture, taste and nutrition while considering sustainability helps to create food that is suitable for the many needs of consumers today. An understanding of how protein–carbohydrate interactions function, the factors that influence them, and their potential applications is considered essential for the development of next-generation foods that are health-promoting, environmentally sustainable, and widely acceptable [15].

Existing reviews on protein–polysaccharide complexes, Maillard conjugates, and starch–protein interactions have generally examined these systems separately [16]. The present review provides an integrative perspective by applying a unified framework based on interaction type × food matrix × processing condition × functionality to compare protein–carbohydrate interactions across different food systems. This framework is further supported by quantitative comparisons and by a focused discussion of AI- and omics-assisted approaches, which are increasingly shifting the field from descriptive observation toward predictive design. In addition, this review distinguishes interactions with established commercial relevance from those that remain largely at the laboratory stage and highlights key safety and regulatory concerns, particularly those related to dietary advanced glycation end products (AGEs).

To operationalize this framework throughout the manuscript, Table 1 summarizes representative protein–carbohydrate interactions across four interconnected dimensions: (i) interaction mechanism (covalent and non-covalent interactions), (ii) food matrix (bakery, dairy, meat, beverages, and plant-based systems), (iii) processing conditions (thermal processing, enzymatic treatment, fermentation, pulsed electric fields, ultrasound, and drying), and (iv) resulting functional outcomes, including texture, stability, digestibility, sensory quality, and nutritional performance. Rather than discussing these variables independently, this review emphasizes how their combined effects determine food quality, technological functionality, shelf stability, and application potential in modern food systems.

## 2. Nature and Types of Protein–Carbohydrate Interactions

Protein–carbohydrate interactions play a fundamental role in determining the structural, nutritional, rheological, and sensory properties of food systems. These interactions occur through both covalent and non-covalent mechanisms and are strongly influenced by factors such as pH, temperature, ionic strength, water activity, and food matrix composition [17]. These interactions dominate the development of brown color, roasted aroma, antimicrobial melanoidins, and long-term shelf-life behavior. Non-covalent interactions (hydrogen bonding, electrostatic attraction, hydrophobic association, and van der Waals contact) are weak, reversible, and respond to pH, ionic strength, and temperature [18]. They modulate functional behavior (solubility, gelation, emulsification, foaming, viscosity) without altering molecular identity [19]. This distinction is the organizing principle of Section 2.1 and Section 2.2 and is summarized in Table 2.

### 2.1. Covalent Interactions

Covalent protein–carbohydrate interactions involve the formation of stable chemical bonds between reactive groups of proteins and carbohydrates. These interactions are generally irreversible under normal food-processing and storage conditions and significantly influence the physicochemical, nutritional, and functional characteristics of food systems. Covalent interactions are particularly important in thermally processed foods, where heat accelerates the reaction between amino groups of proteins and carbonyl groups of reducing sugars. The functional consequences of covalent interactions strongly depend on processing intensity and matrix composition. Controlled covalent modification may improve the solubility, thermal stability, water-holding capacity, and interfacial behavior of proteins. However, excessive reactions may negatively affect nutritional quality by reducing amino acid availability and promoting the formation of advanced glycation end products (AGEs). Therefore, careful control of processing parameters such as temperature, moisture content, reaction time, and pH is essential to optimize the beneficial effects of covalent protein–carbohydrate interactions while minimizing undesirable reactions [20]. Covalent interactions are widely utilized in bakery products, dairy systems, meat analogs, edible films, encapsulation technologies, and plant-based food formulations because of their ability to improve structural stability and technological functionality under complex processing conditions.

#### 2.1.1. Maillard Reaction

**Figure 1:**
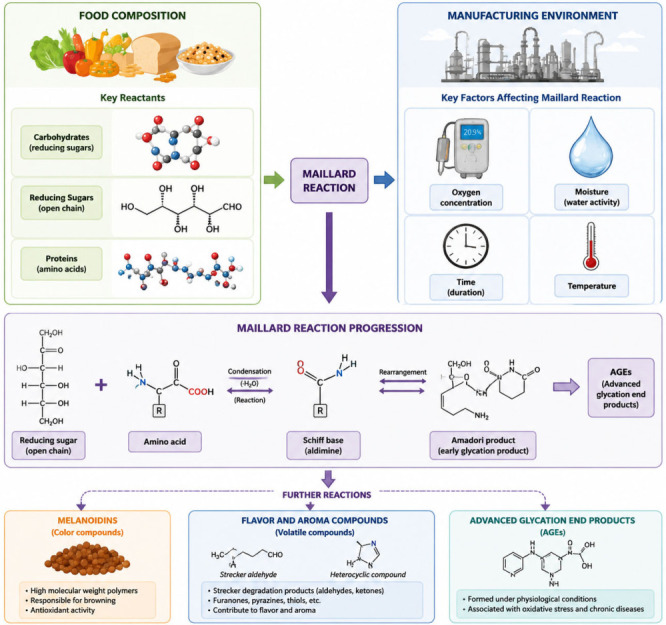
Maillard reaction between proteins and carbohydrates.

The Maillard reaction is one of the most significant covalent interactions occurring between proteins and carbohydrates in food systems. This reaction involves the condensation of carbonyl groups of reducing sugars with free amino groups of proteins, peptides, or amino acids, with lysine being one of the more reactive residues. The reaction is accelerated under thermal processing conditions such as baking, roasting, frying, extrusion, and drying, where elevated temperatures and reduced moisture levels favor the formation of Maillard reaction products [21,22]. The Maillard reaction proceeds through several stages, beginning with the formation of Schiff bases and Amadori compounds, followed by complex rearrangements that generate intermediate and advanced reaction products. These reactions substantially contribute to the development of characteristic flavor compounds, brown pigments, and volatile aromatic molecules in thermally processed foods. Pyrazines, furans, aldehydes, ketones, and thiophenes formed during Maillard chemistry are primarily responsible for roasted, baked, caramelized, and grilled sensory attributes shown in Figure 1 [23].

The extent and functional outcome of the Maillard reaction strongly depend on food matrix composition, temperature, water activity, pH, and processing duration. In bakery and cereal systems, controlled Maillard reactions improve crust color, flavor complexity, and textural properties, whereas excessive glycation may lead to undesirable hardening and nutrient degradation. In dairy and beverage systems, mild glycation can improve emulsion stability and protein dispersibility without causing excessive browning [24,25]. To illustrate some examples, the Maillard reaction in bakery systems is not only associated with the enhancement of the crust color and flavor but also influences the moisture dynamics and crumb softness [26]. Polysaccharides used as functional additives in meat formulations include carrageenan, alginate, and cellulose derivatives such as methylcellulose and carboxymethylcellulose, which enhance textural quality. Native cellulose is rarely used as a direct functional binder and mainly serves as a structural fiber [27]. Protein complexes with carbohydrates are useful in stabilizing emulsions in dairy and plant-based beverages and may also prevent sedimentation or phase separation [28].

Beyond flavor and color development, controlled Maillard conjugation can significantly improve the techno-functional properties of proteins. Early stage glycation enhances protein solubility, emulsifying capacity, foaming behavior, thermal stability, and antioxidant activity by introducing hydrophilic carbohydrate moieties onto protein surfaces. These conjugates are increasingly applied in dairy emulsions, plant-based beverages, edible films, encapsulation systems, and functional foods because they improve colloidal stability and interfacial performance under varying processing conditions [29].

Despite its technological benefits, uncontrolled Maillard reactions may negatively affect nutritional quality. Excessive thermal processing reduces lysine bioavailability and promotes the formation of advanced glycation end products (AGEs), which have been associated with oxidative stress, inflammation, and metabolic disorders. Therefore, balancing the beneficial functional effects of Maillard conjugation while minimizing nutritional deterioration remains a major challenge in modern food-processing [30,31].

In addition, the rate and extent of these interactions also depend upon the nature of the sugar, the source of proteins, temperature, pH, and water activity. For example, in heating conditions, the milk proteins themselves can more easily react with lactose [32]. This presents an issue in the production of infant formula and UHT milk. The Maillard reaction and other protein–carbohydrate interactions therefore need to be controlled to balance flavor formation and nutritional preservation [33].

#### 2.1.2. Enzymatic and Chemical Cross-Linking

In addition to the Maillard reaction, proteins and carbohydrates may also interact through enzymatic conjugation and chemical cross-linking mechanisms. These covalent modifications are widely applied in food systems to improve structural stability, rheological behavior, water-holding capacity, and resistance to environmental stress during processing and storage. Compared with uncontrolled thermal glycation, enzymatic and chemical approaches provide greater specificity and allow for the formation of targeted covalent linkages under relatively mild processing conditions. Enzymatic conjugation commonly involves the use of enzymes such as transglutaminase, laccase, tyrosinase, and peroxidase to promote covalent bonding between proteins and carbohydrate-containing compounds. Among these enzymes, transglutaminase is the most extensively utilized in food applications because of its ability to catalyze acyl-transfer reactions between glutamine and lysine residues, thereby strengthening protein networks and improving gel formation. Enzymatic cross-linking is particularly advantageous because it operates under moderate temperatures and pH conditions, minimizing nutrient degradation while enhancing functional performance [34]. Protein–carbohydrate conjugates produced through enzymatic modification exhibit improved emulsifying activity, thermal stability, foaming properties, and viscoelastic behavior. These improvements are associated with enhanced intermolecular networking and increased steric stabilization within food matrices. Consequently, enzymatically modified systems are widely applied in dairy products, meat analogs, bakery formulations, edible coatings, and encapsulation technologies [35]. Protein complexes with carbohydrates are useful in stabilizing emulsions in dairy and plant-based beverages and may also prevent sedimentation or phase separation [36]. Recent developments in enzyme-assisted conjugation and green processing technologies have increased interest in covalent protein–carbohydrate modification as a sustainable strategy for developing clean-label and functional foods. These approaches enable the production of stable food systems with improved textural, nutritional, and technological properties while reducing the need for synthetic stabilizers and chemical additives [37,38].

### 2.2. Non-Covalent Interactions: Hydrogen Bonding, Electrostatics, and Hydrophobic Forces

Non-covalent interactions are reversible associations between proteins and carbohydrates that occur without the formation of permanent chemical bonds. These interactions are primarily governed by hydrogen bonding, electrostatic attraction, hydrophobic interactions, and van der Waals forces. Unlike covalent interactions, non-covalent associations preserve the chemical identity of the interacting molecules and remain highly sensitive to environmental conditions such as pH, temperature, ionic strength, moisture content, and concentration [39]. The behavior of non-covalent interactions strongly depends on the physicochemical characteristics of the food matrix. Electrostatic interactions are particularly important in oppositely charged protein–polysaccharide systems, whereas hydrogen bonding dominates many starch–protein and fiber–protein interactions. Hydrophobic interactions become increasingly significant in low-moisture and lipid-rich systems, including meat analogs, ice cream, and fat-reduced formulations [40]. In addition to their technological importance, non-covalent interactions may influence nutrient digestibility and bioavailability. Certain protein–carbohydrate complexes can slow enzymatic hydrolysis, regulate nutrient release, and improve the protection of sensitive bioactive compounds during gastrointestinal digestion. These properties are increasingly exploited in the development of functional foods, controlled-release systems, and nutritionally optimized formulations. Modern food-processing technologies, including ultrasound, high-pressure treatment, pulsed electric fields, and controlled fermentation, further influence the strength and distribution of non-covalent interactions within food matrices. Consequently, understanding the mechanisms governing these reversible interactions is essential for designing stable, clean-label, and functionally enhanced food systems [41].

#### 2.2.1. Protein–Monosaccharide Interactions in Food Quality

The electrostatic forces can also be formed when polar groups of sugars are close to charged amino acid residues, namely, lysine or arginine [42]. These interactions can achieve the following effects.

Improve Protein Solubility: Protein aggregation could also be inhibited through the binding of monosaccharides that surround the protein surface with hydration shells that enhance protein solubility in aqueous environments [43]. Carbohydrates interact with proteins to improve solubility, which plays an important role in determining protein functionality in food systems. This hydration shell enhances the interaction between the protein and water molecules, thereby reducing hydrophobic interactions and inhibiting protein aggregation [44].

The hydration shells formed by sugars on proteins provide hydrophobic, protective hydration patches and stabilize the protein’s tertiary structure to keep the proteins in a more dispersed and soluble form. This is especially useful in acidic or high-ionic-strength environments wherein a lot of proteins are apt to cluster or fall out. The solubility is directly correlated with the protein’s capacity to engage in emulsification, foaming, and gelation processes, which are critical in the formulation of stable beverages, dairy alternatives, sauces, and nutrition supplements [45].

Also, the glycation of proteins at controlled stages through the addition of reducing sugars or polysaccharides can produce protein–carbohydrate conjugates which are highly soluble and possess a variety of ideal functional properties for clean-label formulations and high-performance food [46].

Stabilize Emulsions: Sugar can also play important roles in emulsified systems, e.g., salad dressings or dairy emulsions, where protein–sugar interactions are believed to contribute to interfacial stability by increasing the surface activity of proteins. In these systems, e.g., the association of protein with sugar through non-covalent bonding or controlled glycation may greatly augment the surface activity of proteins, leading to improved adsorption at interfaces and reduced surface tension [47]. Proteins may also acquire amphiphilic characteristics when glycated or sugared and may prove to be a more active emulsifying agent. The increase in repulsive forces that accompanies the carbohydrate moieties in both steric and electrostatic interactions also improves the physical stability of the emulsion, particularly in instances of thermal processing, a change in pH or alterations caused due to ion variations. As an example, in the case of dairy systems, lactose interacts with casein or β-lactoglobulin milk proteins to produce enhanced emulsifying characteristics when lactose is heat-treated [48]. In vegetable-based emulsions, analogous enhancements are attained by the glycation of vegetable proteins (soy or pea protein) with dextrins or different polysaccharides.

Modify Mouthfeel and Viscosity: Protein and sugar interactions are one of the non-covalent interactions that may affect the rheology of a product and lead to thickening or creaminess, as observed in protein shakes [49].

Protein–monosaccharide interactions can help stabilize proteins against mild temperature and pH changes during food processing. They also decrease the rate of protein denaturation and precipitation, which enhances shelf-stability. In low-fat or reduced-sugar products, they replace the lost mouth-feel or binding properties, which helps to maintain consumer-acceptable products [7]. Non-covalent interactions between proteins and monosaccharides are subtly but significantly responsible for defining the quality, stability and behavior of food products. They have a wide influence on processing and sensory quality, as well as nutrition; this makes them a potent instrument in modern food design and formulation [50].

#### 2.2.2. Nature of Non-Covalent Protein–Oligosaccharide Interactions

Protein and oligosaccharide non-covalent interactions can include protein and oligosaccharide hydrogen bonding, electrostatic interactions, and van der Waals force and hydrophobic interactions. Oligosaccharides (3–10 monosaccharide units) are rather branched and flexible, with several hydroxyl groups. These oligosaccharides interact with amino acid side-chains through weak reversible interactions, reversible and non-disruptive forces that do not alter the identity of the ingredients. Such interactions are dynamic and sensitive to the environment and dependent on factors such as pH, temperature, ionic strength and concentration [51].

These interactions can achieve the following.

Enhanced Emulsion Stability: The addition of proteins such as protein oligosaccharide complexes increases the stability of oil-in-water emulsions. The oligosaccharide side chains increase the hydration layer around protein molecules, preventing the coalescence of oil droplets. This property is useful in salad dressings, dairy emulsions and nutritional beverages [52].

Improved Foaming and Air Incorporation: Non-covalent complexes can enhance the foaming capacity and foam stability of proteins. Protein surfaces are more flexible and interfacial tension is lower thanks to oligosaccharide additions that allow for the more effective trapping of air, and are useful in whipped toppings and mousses and bakery batters [53].

Viscosity and Gelation Control: Through weak interactions, oligosaccharides modify protein network formation. Such interactions enhance water binding and contribute to increased viscosity and the formation of soft, low-brittleness gels, which is attributed to the presence of high-molecular-weight polysaccharides [54]. This property is particularly useful in protein-based yogurts and puddings.

The non-covalent interactions between proteins and oligosaccharides offer multipurpose potential in food design. These interactions improve both nutritional functionality and technology performance by enhancing emulsion stability, texture, and flavor, as well as appetitive-controlled nutrient release [55]. The reversible nature of these interactions enables food technologists to develop products with tailored properties, including clean-label formulations and potential health-promoting effects.

#### 2.2.3. Nature of Protein–Polysaccharide Non-Covalent Interactions

Polysaccharides are complex carbohydrates that are long chains of monosaccharide units, which attach to proteins mainly by means of non-covalent interactions, hydrogen bonding, electrostatic interactions, hydrophobic interactions, and van der Waals forces [56]. Such interactions will depend on the molecular weight, charge density, branching, and solubility of the polysaccharide, and the pH, ionic strength, and temperature of the food matrix.

Non-covalent protein–polysaccharide complexes may reduce enzymatic degradation by shielding active sites. These may slow down digestion and generate slowed delivery systems for nutrients, which are healthy in terms of metabolism and controlling long-term energy capacity [57]. They safeguard labile nutrients (e.g., vitamins, antioxidants) against losses during processing. Other polysaccharides, e.g., alginate or pectin, may escape complete digestion in the upper GI tract but are fermented in the colon. During interactions with proteins, which occur non-covalently, these could provide delivery vehicles that coordinate the release of bioactive (e.g., peptides, phenolics) in the large intestine to facilitate gut health and targeted absorption [58].

Polysaccharide and protein interactions are mainly non-covalent interactions that are used to structure and stabilize food systems. These interactions enable the fine-tuning of the product formulations to ensure the desired quality and functional performance by establishing networks, water retention, and emulsification, and by regulating nutrient delivery. These are flexible and, therefore, are invaluable in the development of contemporary foods that are healthy and presentable and will have an unrestrained shelf life.

**Figure 2:**
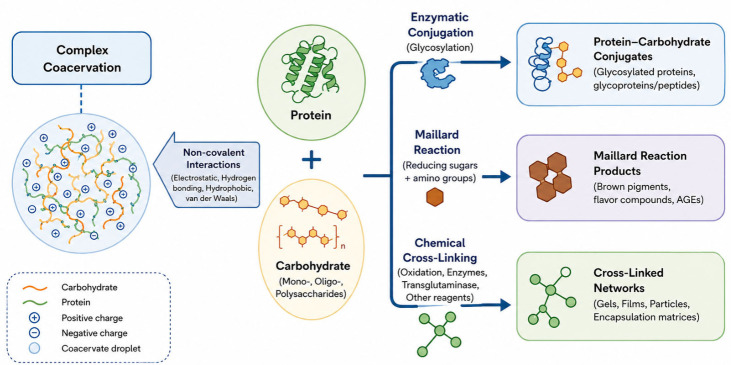
Pathways of protein–carbohydrate interactions, including non-covalent association, complex coacervation, enzymatic conjugation, Maillard reaction, and chemical cross-linking, leading to functional complexes for stabilization, texture, and encapsulation.

Although covalent bonds last, non-covalent bonds provide flexibility, which can be changed, and are reactive to conditions like pH, the number of ions and the temperature. Protein–polysaccharide systems are formed through both non-covalent and covalent interactions. However, substances spontaneously form non-covalent interactions such as electrostatic interactions, hydrophobic interactions, hydrogen bonds and van der Waals forces when mixed, and these can help in the formation of emulsion stabilizers, gels, thin films and edible protective coatings. In general, most non-covalent interactions are weak and readily reversible and can be modulated or stripped away by tuning the pH, ionic strength, temperature, and so on [59]. Covalent interactions through the Maillard reaction, enzyme-catalyzed reactions and chemical cross-linking reactions build covalent bonds and make protein–polysaccharide complexes more stable [60]. However, to obtain this reaction, it is necessary that reaction conditions like temperature, pH, ionic strength and reaction time are modified [61]. Figure 2 summarizes the principal pathways involved in protein–carbohydrate interactions, including non-covalent association, complex coacervation, enzymatic conjugation, the Maillard reaction, and chemical cross-linking.

These four modes differ in their bond character and processing requirements, as follows: (i) physical co-mixing relies on spontaneous non-covalent contacts (electrostatic attraction, hydrogen bonding, hydrophobic association, and van der Waals forces) and forms readily upon simple blending, producing reversible emulsion stabilizers and gels; (ii) complex coacervation is a charge-driven associative phase separation between an oppositely charged protein and polysaccharide, used for microencapsulation and colloidal stabilization, and is tuned by pH and ionic strength; (iii) enzymatic conjugation uses transglutaminase or related enzymes to form targeted covalent links under mild conditions, improving thermal stability with minimal nutrient loss; (iv) the Maillard reaction forms covalent C–N bonds between reducing-end sugars and protein amine groups under heat, providing the most stable conjugates but carrying the nutritional trade-offs discussed in Section 2.1. Section 2.2.4, Section 2.2.5 and Section 2.2.6 examine the dominant non-covalent forces among these modes.

#### 2.2.4. Hydrogen Bonding

**Figure 3:**
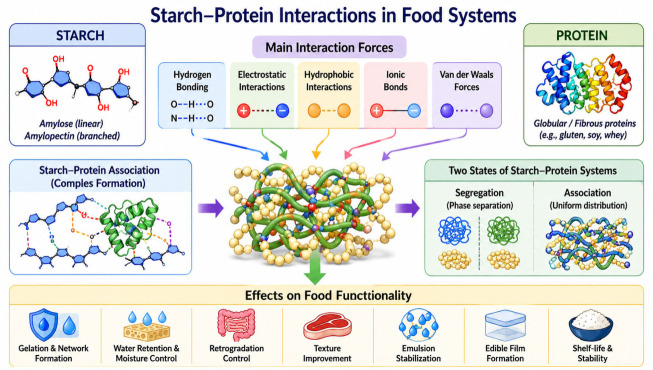
Starch–protein interactions in food systems, showing hydrogen bonding, electrostatic and hydrophobic interactions, and covalent modifications, and their effects on gelation, texture, and retrogradation.

Hydrogen bonds form between polar groups on amino acids (OH, NH_2_) and hydroxyl groups of polysaccharides, improving the uniformity and stability of food gels, emulsions, and films [62]. Hydrogen bonding is the dominant interaction in protein–pectin and protein–cellulose systems, with up to 14 hydrogen bonds being formed during cellulose–myosin binding, thereby contributing to texture and moisture retention in meat products [63]. Protein–starch systems exist in two states: segregation (phase separation due to thermodynamic incompatibility) or association (an even distribution with direct interactions). These states strongly influence structural and functional properties. The main interaction forces, covalent bonds, electrostatic interactions, hydrogen bonds, ionic bonds, hydrophobic interactions, and size differences are illustrated in Figure 3**.**

#### 2.2.5. Electrostatic Interactions

The interaction between amino acids and polysaccharides (e.g., alginate, pectin) could be achieved through the charged group, which has a major effect on stabilizing emulsions, forming coacervates and microencapsulating various substances [64]. An example is that polyphenol–protein–sugar complexes, obtained mainly by electrostatic forces, could make milk–tea emulsions better at foaming and maintain colloidal stability. The presence of SLF milk protein complexes makes milk thicker and more stable by attracting each other through electrostatic and hydrogen bonding. The presence of SLF is explains in Section 7.2.

#### 2.2.6. Hydrophobic Interactions

When polysaccharides or modified carbohydrates have non-polar regions, these regions can interact with the non-polar parts of proteins through hydrophobic forces. Interactions at these levels mostly take place in systems that are low in moisture and emulsified with oil [49]. In systems where lupine protein is bonded with milk fat mimetics, this helps ice cream remain solid and feel more pleasant to eat, and maintains its airy texture. These interactions in meat analogs help the matrix bonds and provide a meat-like mouthfeel.

## 3. Structural Implications of Protein–Carbohydrate Interactions

When interpreted within the four-dimensional framework in Table 1, the systems below differ mainly in the food matrix and processing conditions, rather than in interaction chemistry, which is why similar bond types produce different functional outcomes across products

The structural characteristics of many food products are strongly governed by the way proteins and carbohydrates interact. These macromolecular interactions play an important role in network formation, gel strength, and retrogradation processes [3]. This could protect the system microstructure and be important for food texture, water-holding capacity, strength and shelf life. Modifying the structure through the interactions between proteins and carbohydrates helps to determine the properties of food during cooking, storing and eating.

Overall, the behavior of protein–carbohydrate systems depends not only on the interaction mechanism itself but also on the surrounding food matrix and processing conditions. Covalent interactions, particularly Maillard conjugation, are more dominant in thermally processed bakery, dairy, and roasted food systems, where they contribute to flavor development, browning, emulsification, and shelf stability, although excessive reactions may reduce nutritional quality through AGE formation. In contrast, non-covalent interactions are more important in hydrated and colloidal systems such as beverages, dairy emulsions, meat analogs, and edible films, where hydrogen bonding, electrostatic attraction, and hydrophobic interactions regulate gelation, water retention, viscosity, and emulsion stability. Processing technologies including fermentation, enzymatic modification, ultrasound, and pulsed electric fields further influence the balance between covalent and non-covalent interactions, thereby determining the final structural, sensory, and nutritional functionality of food products. These comparisons highlight the matrix-dependent and process-dependent nature of protein–carbohydrate interactions in modern food systems.

### 3.1. Network Formation and Gelation

Three-dimensional structures in food are built by combining proteins and carbohydrate molecules. These are usually formed through non-covalent means, including hydrogen bonding, electrostatics and hydrophobicity, and sometimes through covalent means such as Maillard-type connections or conjugation with enzymes [4]. Examples of this include soy protein with rice starch and corn gluten with potato starch, which have shown increased storage modulus (G′) and loss modulus (G″) in viscoelastic testing, suggesting these mixtures are more rigid and can resist changes in shape [65]. When fibers like pectin or β-glucans are present in proteins, hydrogen bonding pulls them together to form a gel, which improves the amount of water the product can hold and makes the texture more stable in low-moisture conditions. With fermented materials such as okara, bioconversion with microbes helps the protein and fiber components gel better, creating materials with better cohesion and antioxidant properties.

### 3.2. Starch Retrogradation and Textural Softness

Starch retrogradation is when gelatinized starch (mainly amylose) starts recrystallizing as the product is cooled [66]. This sets up bread and cooked rice to become firmer and less tender as they age. How proteins interact with carbohydrates can cause retrogradation to occur in one case and prevent it in another. Rice protein hydrolysates and whey protein isolates were reported to bind amylose [67], join to the amylose molecules in rice noodles and bread and prevent them from forming hard crystals, ensuring that these foods remain soft after steaming. On the other hand, there are plant protein additives that, depending on the pH and ion concentration in the dough, may assist in forming tight complexes with amylose. These can increase the firmness of the baked item. Over time, this may also lead to increased water loss. The process of retrogradation is influenced by Maillard conjugates. These conjugates can alter starch re-crystallization, which affects the texture and shelf life of baked goods.

A small amount of glycation helps starch and proteins stay flexible and soluble, but too much leads to stiffness and loss of solubility [68].

### 3.3. Microstructural Cohesion and Film Formation

The formation of edible films and coatings used in food packaging, moisture barriers and active release systems is mainly possible due to interactions between proteins and carbohydrates. As a result, whey protein starch films show greater resistance to oxygen, more flexibility and stronger barriers. Because of these qualities, they are perfect for maintaining freshness in bakery and cheese items [69]. When oat protein isolate and *Pleurotus ostreatus* β-glucan are Maillard linked, the result is a thermally stable and porous gel with better construction, which is ideal for use in processed and practical food products. A combination of hydrophilic carbohydrates, which regulate moisture, and protein-based cross-links, which make the films stronger, benefits these films [70].

### 3.4. Impacts on Processed and Plant-Based Systems

The integration of fiber and protein in processed meat and analogs (e.g., pectin and myosin, cellulose and actin) strengthens texture, avoids the formation of separate layers and allows the product to hold more moisture during cooking [71]. These are especially necessary when meals are designed to be high in protein and low in fat, since both texture and juiciness are often affected. For ice cream, changing skim milk powder to sweet lupine flour caused the ice cream to keep its shape during melting, have better foaming properties and be creamier, thanks to its protein–carbohydrate content. Mixing rice starch with corn or soy proteins in food that avoids gluten helps the dough retain its shape and makes it stick together, which is crucial for products like noodles, pizza crusts and flatbread. Protein–carbohydrate interactions are key to the structure of modern foods. These promote gel formation, prevent components from deforming, reinforce edible films, and stabilize emulsions [7]. This kind of interaction gives food developers control over the mechanics and ensures the product maintains its improved properties in different situations. By using strategic approaches and improving processing, these interactions lead to the creation of clean-label, long-lasting and effective foods that please consumers.

## 4. Nutritional Properties of Protein–Carbohydrate Interactions

Any changes in how proteins and carbohydrates interact in foods usually affect their nutritional value, mainly through digestion, which could influence blood sugar and the absorption of micronutrients. These occur by introducing physical obstacles and causing chemical changes in nutrients meaning that the body can no longer process them effectively [72].

The cooking quality of grains largely depends on starch gelatinization and thermal stability [73]. The way starch ages during storage can change the texture of grains after they have been cooked. How food made with starch tastes and feels depends greatly on its gel-like structure.

Proteins can interfere with the enzymatic breakdown of starch, particularly by inhibiting the activity of α-amylase, the enzyme responsible for starch hydrolysis in products such as noodles. When suitable proteins are added to starchy foods, they can form complexes with starch molecules, thereby reducing starch digestibility. This modification enhances the functional properties of the resulting food products, particularly by improving glycemic control and promoting satiety [74]. The digestibility of starch is also influenced by thermal processing, chemical treatment, and enzymatic action during digestion. These factors collectively determine the nutritional and functional benefits of starch-containing foods [75]. For example, enzymatic activity plays a crucial role in the development of low-digestibility grains, which are increasingly valued in health-focused food formulations.

**Figure 4:**
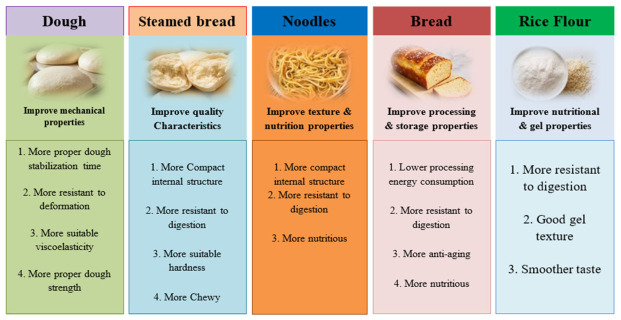
Application of protein–carbohydrate mixtures in starchy food.

Recent studies have shown that incorporating proteins into wheat gluten and starch-based matrices can significantly alter the structural and functional properties of various food products, including dough, fresh bread, fully baked bread, noodles, and rice flour. These modifications can affect texture, shelf life, and nutritional profiles (Figure 4).

### 4.1. Modulation of Digestibility and Glycemic Response

One of the main points highlighted in protein–carbohydrate interactions is their role in carbohydrate degradation. Aggregates could be formed through the interaction between starch and protein, which would limit the digestion of digestive enzymes, such as α amylase. With less glucose being released, the GI goes down and hunger is reduced for longer. Rice protein hydrolysates could attach to starch, which makes its structure more orderly and prevents starch hydrolysis in noodles and steamed bread [76]. The consumption of soy and whey proteins is believed to reduce the rate of starch digestion by forming complexes that resist enzymatic breakdown. This effect can lead to an increase in resistant starch content, which may help regulate blood glucose levels and support weight management in individuals with diabetes.

In the case of functional foods, nutrients could be slowly released during digestion through interactions between different ingredients. A steady supply of energy is provided in sports nutrition and clinical formulas when both protein and carbohydrates are consumed together, without causing excessive insulin response [77].

### 4.2. Bioavailability and Nutrient Losses

However, these interactions may also reduce nutrient bioavailability under certain conditions, even if protein–carbohydrate interactions can lower blood sugar levels. As discussed in Section 2.1.1, uncontrolled Maillard reactions reduce lysine availability and form advanced glycation end products (AGEs) [78,79]. Furthermore, certain combinations of dietary fibers and proteins (e.g., cellulose and myosin) can bind tightly, which supports protein stability but may reduce the rate of enzymatic proteolysis in meals [80].

### 4.3. Controlled Glycation and Functional Benefits

Mild, controlled glycation produces protein–carbohydrate conjugates that retain their nutritional value while gaining functional advantages over native proteins. Reaction parameters (temperature typically 50–80 °C; water activity 0.4–0.7; reaction times of hours rather than minutes) are chosen to drive the early Maillard stage (Amadori product formation) without progressing to advanced glycation end products. Three functional outcomes are consistently reported:

(i) Solubility: Oat protein conjugated with β-glucan or pea protein conjugated with maltodextrin show improved solubility across a wider pH range, including the isoelectric region, where the native protein is poorly soluble [81].
(ii) Emulsifying Capacity: The carbohydrate moiety provides steric and electrostatic stabilization at the oil–water interface, increasing emulsion stability under thermal and pH stress [82].
(iii) Antioxidant Activity: Amadori products and early melanoidins contribute to radical-scavenging activity, which is useful in oxidatively unstable matrices such as plant-based beverages and infant formula [83].

These conjugates are increasingly used in clinical nutrition, sports nutrition, and products targeted toward the elderly, where high solubility, mild flavor, and antioxidant carryover are simultaneously required.

### 4.4. Role of Fiber and Plant Proteins

References [14,84] found that adding SLF to low-fat ice cream increased its antioxidant content and nutritional value while lowering its glycemic index. Improved connections between milk proteins and SLF components allowed for syneresis. Additionally, in the case of milk–tea mixes and enriched bread, combining polyphenols, proteins, and carbohydrates can help maintain the bioactive components and increase the food’s health benefits [14]. In terms of food nutrition, these interactions can have both positive and negative effects. When they are properly managed, foods become denser in protein, higher in antioxidants, and lower in their glycemic index, all of which enhance metabolism and increase feelings of fullness. Uncontrolled glycation can reduce the availability of amino acids and contribute to the production of toxic compounds. To create nutrient-dense and healthful food systems, innovative work should focus on simplifying the ways in which fermentation, enzymes, and processing impact these interactions [81,83].

## 5. Sensory and Functional Properties

A food’s structure, nutrition, flavor, aroma, color, and mouthfeel, and key characteristics including emulsifying, foaming, and water-holding, are all influenced by the interaction between protein and carbs [85]. These characteristics are crucial for food acceptance and market success, particularly for processed, plant-based, and clean-label goods.

### 5.1. Flavor and Aroma Development

As established in Section 2.1, Maillard chemistry generates pyrazines, furans, aldehydes, and thiophenes that account for roasted, baked, and grilled aromas [86]. In dairy, baked goods, and meat analog systems, the focus shifts from understanding the mechanism itself to optimizing the extent of the reaction: it should be sufficient to deliver desirable sensory notes, but not so extensive as to promote AGE formation or lysine loss.

### 5.2. Modulation of Perception of Taste

Protein–carbohydrate interactions can improve the perception of sweetness, enhance umami characteristics, and reduce bitterness in plant-based foods [7]. By altering proteins in this way, individuals can enjoy novel, high-protein beverages or meat substitutes because they taste better and feel creamier. The Maillard reaction bonding of oat protein isolate with β glucan enhances emulsification and flavor retention in emulsions, as shown in Table 3.

### 5.3. Texture and Mouthfeel Enhancement

Water-absorption capacity, lubrication behavior, and gel viscosity collectively influence mouthfeel by determining lubrication efficiency, perceived thickness, and overall sensory perception during consumption. According to research, glycated proteins produce thicker and more cohesive emulsions and foams, which are even more apparent in low-fat and dairy-free goods. Whey protein–pectin coacervates increase gel storage modulus G’ at pH 4.0 [90]. Stable protein–carbohydrate networks formed in sweet lupine flour ice cream (Section 7.2) demonstrate the relationship between glycation, foam structure, and creaminess [91].

### 5.4. Color and Visual Appeal

Browning is a result of the Maillard process and is frequently associated with a product’s freshness and flavor. Foods like crusty bread, grilled meat, and caramelized syrups are typically seen as more cooked and delicious when they turn brown [92]. However, there are instances in baking, including dairy and white bread goods, where excessive browning could be considered undesirable. The food-processor may produce goods with the required colors by adjusting temperature, pH, and moisture [93]. Optimal crust browning corresponds to an L* value of around 50–60, measured by CIE colorimetry [94]

Emulsification, Foaming, and Stability: The interfacial and colloidal effects brought about by the coupling between proteins and carbohydrates are primarily responsible for the good performance of food products.

Emulsification: Glycated proteins create interfacial sheets that prevent oil and water emulsions from combining and separating. Making mayonnaise, dairy beverages, and sauces requires whisking. PEF-assisted BSA–starch conjugates achieved an emulsifying activity index of roughly 36.00 m^2^/g compared to 18.00–22.00 m^2^/g for native BSA.

Foaming: When the hydrophilic hydrophobic balance of proteins is adjusted, air incorporation and foam stability, both essential for whipped toppings and mousses, are increased [95].

Water-Holding: A network of proteins and carbs helps baked foods and meat substitutes retain water, extending their shelf life and improving their texture [96]. Protein–carbohydrate interactions improve both sensory quality and functional performance in food systems. The way chemicals interact to affect flavor, aroma, texture, and moisture guarantees that customers will enjoy the product and that it will function well. Food scientists create high-quality, well-liked, and visually appealing food products by using regulated procedures, various ingredients, and enzymes or microbes.

## 6. Role of Processing Techniques in Protein–Carbohydrate Interactions

Food-processing techniques have a significant impact on how proteins and carbohydrates react to one another. The shape and function of protein–carbohydrate complexes are altered by heat-processing, enzymatic modifications, fermentation, and contemporary techniques, including pulsed electric fields (PEF) and ultrasound. Understanding and managing the impacts on the nutritional, structural, and sensory qualities is crucial for creating healthier food systems [33].

### 6.1. Thermal Processing

Thermal processing controls the extent of the Maillard chemistry described in Section 2.1, which is mostly brought on by heat treatment, changes the structure of proteins and provides flavor, color, and emulsification. Food is heated and dried during baking, roasting, and extrusion to stimulate Maillard browning, which improves flavor and texture. However, excessive thermal processing can reduce protein digestibility and promote the formation of AGEs [97]. It is important to avoid overprocessing, overheating, and loss of nutrients and quality during thermal processing in low-moisture systems such as bakeries and confectioneries.

### 6.2. Enzymatic Modification

Protein solubility, emulsifying ability, digestibility, and the way proteins interact with polysaccharides are all frequently enhanced by enzymatic hydrolysis. A study by [98], showed that utilizing enzymes to degrade plant proteins produces peptides that are effective at emulsifying and binding water, which aids in the formation of stable protein–carbohydrate complexes. The rheology of gluten-free dough is positively impacted by binders formed from enzymatically hydrolyzed rice or lupine proteins, which more readily cling to starch or inulin and slow down retrogradation [99]. Since high solubility and emulsification are essential, this method is particularly suitable for beverages, baby food, and meat alternatives

### 6.3. Microbial Fermentation

Protein–carbohydrate matrices can be made stronger, more digestible, and more bioactive by employing the sustainable fermentation process [100]. Reference [101] demonstrated that by altering the protein and polysaccharide levels, fermenting okara improved its antioxidant qualities and gel strength. Fermentation improves the body’s ability to interact with food products like plant-based yogurts and healthy drinks by lowering anti-nutritional factors, increasing prebiotic content, and changing the molecular structure.

### 6.4. New Technology for Processing

Pulsed electric field (PEF) processing involves the application of short, high-voltage pulses. By altering the structure of proteins and dissolving the cell membrane, these pulses increase the number of interaction sites for the binding of carbohydrates [102]. Because the overall impact and heat-sensitive vitamins can be preserved, this is particularly useful for low-heat processing.

### 6.5. Ultrasound and High-Pressure Processing

These techniques help carbohydrates adhere to proteins more effectively, disrupt protein clumps, and reveal hydrophobic and charged areas that are otherwise hidden. Because these reactions are easier to regulate, emulsions with antioxidant properties can be produced quickly [103].

### 6.6. Drying and Dehydration

Food is dried using techniques including vacuum-drying, spray-drying, and freeze-drying, which eliminate water, altering the interactions between proteins and carbohydrates and potentially causing aggregation or the creation of films [104]. Protein–pectin matrices and whey starch films operate differently as a result of drying procedures. Compared to thermal-drying, vacuum-drying preserves more antioxidants and produces a higher-quality microstructure [105]. The substances that give matrices their biological and functional qualities are not harmed if they are dried properly.

### 6.7. Ingredient Formulation and Matrix Design

Processing decisions should align with ingredient selection for optimal outcomes. When skim milk powder is substituted with sweet lupine flour in low-fat dairy products and processed under controlled conditions, such as pasteurization and moderate homogenization, the foam’s retention, melting speed, and antioxidant activity are all improved. In meat analogs, fiber components like cellulose and pectin work with myosin and actin to improve the products’ softness and water-holding capacity, particularly during cooking or extrusion [106]. Proteins and carbs can be treated in a variety of ways, both conventional and cutting-edge, to accomplish certain nutritional and functional objectives. Food technologists can improve the quality, longevity, and health of food products by utilizing process control and comprehending how ingredients interact [107].

Across different food matrices, processing conditions strongly influence the balance between covalent and non-covalent protein–carbohydrate interactions and therefore determine the final functionality of the product. Thermal processing in bakery and dairy systems mainly promotes Maillard conjugation, contributing to flavor, browning, emulsification, and shelf stability, whereas non-thermal technologies such as pulsed electric fields, ultrasound, and controlled fermentation primarily enhance non-covalent interactions while minimizing nutrient degradation. Similarly, enzymatic modification and drying techniques alter protein accessibility, hydration behavior, and network formation differently depending on whether the system is starch-based, protein-rich, or fiber-enriched. These comparisons demonstrate that the technological performance of protein–carbohydrate systems cannot be explained by interaction type alone but must also be interpreted within the context of matrix composition and processing intensity.

## 7. Applications in Food Systems

Making use of the links between protein and carbohydrates has led to the development of numerous novel recipes for meat products, dairy substitutes, baked goods, functional beverages, and gluten-free goods. They help foods become more nutrient-dense, improve texture quality, and make food more stable and long-lasting [108]. Emulsifiers can be utilized for plant-based, low-fat, and clean-label products, where consumers prefer natural products without chemical additions [109].

### 7.1. Meat Products and Analogs

Protein–carbohydrate interactions play a major role in the texture, juiciness, and water retention of both conventional and plant-based meat. Reference [110] shows how pectin and cellulose use hydrogen bonds and van der Waals forces to adhere to flesh proteins including myosin, actin, and collagen. These decrease the amount lost during cooking, enhance the product’s feel, and increase the emulsion’s stability. By using these complexes, restructured meats maintain their cohesiveness and firmness, particularly when yarns or fruit and vegetable byproducts like tomato pomace, pineapple, or apple are added. A combination of proteins and carbohydrates, primarily fiber, inulin, or starch, is created in meat analogs, improving the food’s texture, chewiness, and sense of dampness.

### 7.2. Dairy and Plant-Based Dairy Alternatives

Protein–carbohydrate interactions are essential for both dairy-free and milk-based formulations to ensure a thick consistency, frothy bubbles, effective emulsification, and improved nutritional absorption. SLF, which can form a connection with milk proteins and help the product resist melting, increase antioxidant activity, and improve its flavor [111], is used in low-fat ice cream in the place of skim milk powder. Compared to conventional techniques, this invention enables a 30% reduction in production costs. SLF-containing formulations increase the number of phenols, improve the foam’s stability, and give ice cream and toppings a creamy texture. The addition of fiber and polysaccharides, such as guar gum or β glucan, to the legume proteins in nondairy yogurt or Labneh enhances gel strength, prolongs the shelf life of the probiotics, and keeps the product stable.

### 7.3. Bakery and Cereal-Based Systems

Protein–carbohydrate interactions contribute to dough structure, a crisp crumb texture, and the avoidance of staling while baking breads and other products. Protein–starch complexes, such as amylose and wheat gluten, control retrogradation, lessen staling, and enhance product suppleness over extended storage [93]. The Maillard reaction between sugar and amino acids during baking gives breads and cookies their color, aroma, and depth of flavor [112]. The interactions between polyphenols and proteins and starch in bread prepared with tea extracts or fruit polyphenols improve antioxidant effects, stabilize dough, and extend shelf life [113]. The structure, texture, and mouthfeel of gluten-free baked goods are enhanced by the use of proteins like whey or soy isolates and starches like rice, corn, or quinoa.

### 7.4. Functional Beverages and Emulsions

Protein–carbohydrate complexes aid in the stabilization, viscosity adjustment, and release of the active ingredients in beverages [114]. Protein and polyphenols in milk–tea beverages create stable emulsions that adhere to the sugars, preserving stability and enhancing flavor [115]. Additionally, they maintain the aroma for a longer period of time, improve oxidative stability, and guarantee that the liquid stays stable, all of which are critical for plant milks, sports drinks, and fortified smoothies. In clean-label goods, conjugates made via enzymatic cross-linking or the Maillard reaction aid in the formation of emulsions rather than the use of synthetic surfactants.

### 7.5. Edible Films, Coatings, and Food Packaging

Combinations of protein and carbohydrates are increasingly being used in the creation of environmentally friendly packaging. Whey protein–starch films provide food items with their strength, stop moisture loss, and guarantee a controlled release of antioxidants or flavorings [116]. The combination of oat protein and β-glucans, which are linked to Maillard processes, contributes to the films’ flexibility, heat resistance, and retention of larger active chemicals [117]. Because the biopolymer films are biodegradable, they complement the food industry’s environmentally beneficial practices.

### 7.6. Nutraceutical and Functional Food Systems

Blood sugar control, digestion regulation, and effective nutrient delivery are made possible by the inclusion of protein and carbs in functional foods [118]. A key component of medical nutrition and sports nutrition, the progressive release of energy-producing amino acids and starch is made possible by purposefully altering protein–carbohydrate chemical reactions. Dietary fibers like cellulose or pectin, when added to high-protein diets, reduce the glycemic effect, make you feel full, and lessen the time food spends in the stomach, all of which are important metabolic-management objectives [119]. In products like plant-based burgers, low-fat dairy, clean-label emulsions, and edible packaging, food firms employ protein–carbohydrate interactions. These make foods better in several areas, such as their ability, nutrition, how long they keep and how much people enjoy them. Because these interactions can be used in many ways, they play a key role in creating sustainable, effective and forward-looking food systems.

Collectively, these applications demonstrate that the functional performance of protein–carbohydrate interactions is highly dependent on the relationship between the interaction mechanism, the food matrix composition, and the processing strategy. In meat systems, protein–fiber interactions primarily improve water retention and textural stability, whereas in dairy and beverage systems, protein–polysaccharide complexes mainly contribute to emulsion stability, viscosity control, and foam formation. Bakery and cereal products rely heavily on starch–protein interactions and controlled Maillard reactions to regulate retrogradation, softness, flavor, and shelf life. Furthermore, modern processing approaches such as fermentation, enzymatic treatment, and non-thermal technologies allow these interactions to be selectively optimized for targeted nutritional and sensory outcomes. These comparisons highlight the importance of integrating interaction type, matrix environment, processing condition, and functionality when designing next-generation food systems.

## 8. Challenges and Limitations

Despite the fact that protein–carbohydrate interactions significantly enhance food quality, there are still a number of obstacles in the areas of technology, nutrition, and function. Foods present challenges because of their intricate structure, the various suppliers involved, processing challenges, and health issues. Food scientists must have a thorough understanding of these limitations in order to modify protein–carbohydrate interactions without compromising food safety, flavor, or nutritional value.

### 8.1. Complexity of Food Matrices

There are proteins, carbs, fats, water, salts, polyphenols, and micronutrients in actual food systems. Certain protein–carbohydrate interactions are hard to predict and control because of this variety of forms [120]. The behavior of molecules can be readily altered by changing the ionic strength, pH, or moisture content of a solution, which can impact the solubility and the degree of molecular interaction [121]. Additionally, polyphenol or lipid collisions with proteins or carbs may prevent the desired interaction between the proteins and carbohydrates, leading to unanticipated textural changes, layer separation, or decreased stability, as shown in Table 4.

### 8.2. Undesirable Reactions and Nutrient Loss

In addition to giving food its flavor and appearance, the Maillard reaction promotes the development of AGEs in situations involving high heat and low moisture [127]. They are linked to detrimental illness levels that cause oxidative stress, inflammation, and conditions including diabetes and heart issues, in addition to preventing the body from using vital amino acids. Over-processing reduces the health benefits of foods by removing several components, primarily heat-sensitive vitamins and polyphenols [128].

### 8.3. Reduced Digestibility

Strong protein–carbohydrate networks may make it difficult for enzymes to break down protein and starch, which slows down digestion. This is particularly apparent in highly glycated substances and high-density matrices [129]. The complex of cellulose and myosin exhibited a high binding energy and numerous hydrogen bonds, which enhanced the stability of the structure but also increased its resistance to proteolysis [130]. Given the importance of energy and amino acids in senior nutrition, the issues with bioavailability are concerning.

### 8.4. Ingredient Variability

The way that functional foods interact with the intestine can be affected by the different proportions of plant protein, fiber, and polysaccharide branching [131]. Each legume has a unique set of emulsifying and gelling properties that might change depending on the growth environment and processing method [91]. The degree of polymerization and branching in fibers like inulin and pectin also affects their solubility, reactivity, and strength. This makes it difficult to standardize the procedure to achieve consistent results in large production runs.

### 8.5. Processing Challenges

Product quality may not be entirely constant if the technology used struggles to control crucial factors like time, temperature, or humidity [126]. While undercooking can cause food to lose its texture and structure, excessive heat-processing can destroy nutrients. Although they need a large financial commitment and skilled personnel, some more recent techniques, such as ultrasound and pulsed electric fields, might be effective.

### 8.6. Regulatory and Consumer Acceptance

People may be concerned about changed or engineered ingredients if biopolymer engineering makes food systems more complex, and this could affect their decision to purchase particular meals [132]. Maintaining clean-label attributes when employing biotech techniques like fermentation or enzymatic coupling is one challenge. Furthermore, the capacity to employ innovative protein–carbohydrate complexes at a global scale may be impacted by regional variations in safety and approval regulations. Protein–carbohydrate reactions have several advantages, but these are limited by their complex formulation, the unpredictability of the reaction’s outcome, nutritional loss, and other technological difficulties. Experts in food chemistry, process engineering, sensory science, and nutrition must collaborate to address these issues. Resolving these issues and optimizing protein–carbohydrate combinations in modern food manufacture requires the use of sophisticated analytical techniques, cautious processing, and modeling predictions.

## 9. Future Directions

Protein–carbohydrate interactions continue to garner attention as food science advances and consumers seek out healthier dietary options. Even though this discipline has made significant progress, integrating several fields, sourcing responsibly, and utilizing cutting-edge technologies are key to the future. Future food design will greatly benefit from the tools and technologies being created in bioinformatics, omics, and predictive food design, which are helping to provide new insights into these relationships [133].

### 9.1. Integration of Omics and Bioinformatics

Researchers may now examine and comprehend protein–carbohydrate interactions at the molecular level thanks to omics tools such as proteomics, glycemic, and metabolomics.

Reference [134] examined how pectin and cellulose connect to collagen, myosin, and actin in meat using bioinformatics techniques, observing that the interaction is uneven in terms of H bonds and binding force. Scientists can fine-tune recipes for specific results by modeling and predicting how ingredients interact and mimic effects using molecular docking, machine learning, and systems biology. Additionally, these techniques can aid in material selection and improve understanding of the behavior of plant-based proteins and fibers during processing and in byproducts.

### 9.2. Predictive Modeling and AI in Food Design

AI methods enter food formulations at three distinct levels:

I. Molecular Properties Prediction: Graph neural networks trained on glycoprotein structure databases predict binding affinity and conjugation site preference for protein–carbohydrate pairs, narrowing the experimental search space before bench work [135].
II. Process Optimization: Bayesian optimization and reinforcement learning identify temperature, pH, and ionic-strength setpoints that maximize emulsifying activity or gel strength while minimizing AGE formation, using small-batch experimental feedback rather than full factorial designs.
III. Sensory and Nutritional Co-Optimization: Multi-objective machine learning models combine consumer panel data with chemical composition to suggest formulations that simultaneously achieve the target glycemic response and fat content, and have an acceptability score.

Practical bottlenecks remain: most public datasets are small (<1000 entries), cover only a narrow set of matrices, and lack standardized assay protocols. Progress depends on open data-sharing across the food-science community and on hybrid models that combine first-principles food chemistry with data-driven priors. The most promising near-term applications are the reformulation of clean-label emulsifiers, design of personalized low-glycemic carbohydrate blends, and screening of upcycled raw materials.

The industrial adoption of PEF-assisted glycation requires four engineering problems to be solved: energy input per kilogram of product, electrode fouling under high-protein conditions, batch-to-continuous transition, and reproducibility of conjugation degree across runs.

### 9.3. Sustainable and Upcycled Ingredients

Future methods will increase the sustainability and utility of protein–carbohydrate systems by including repurposed materials such okara, wasted grains, and fruit pomace. Fermented okara showed improved gelation and functional properties due to stronger interactions between its proteins and fibers, especially in the presence of added byproducts rich in polysaccharides, reactive proteins, and polyphenols, which may be optimized to enhance bioactivity. Both circular economy and zero-waste concepts are well suited to this type of design.

### 9.4. Emerging Processing Technologies

More control over the behavior of the protein and carbohydrate components is possible due to new technical techniques for precise processing and low temperatures [111]. To alter food macromolecules and preserve nutrients, experiments are being conducted using low-temperature vacuum-drying, cold plasma, ultrasound, and PEF. These procedures are universally helpful in therapeutic settings, newborn nutrition, and the production of functional drinks since they reduce the possibility of the formation of hazardous AGEs, improve the food’s digestive qualities, and preserve its beneficial qualities.

### 9.5. Functional Targeting and Personalized Nutrition

In the future, customized functional meals will provide precise protein–-carbohydrate combinations to address specific health issues, such as foods that are low in glycemic index for diabetics, and senior-friendly foods and beverages enhanced with protein–prebiotic-rich foods for gut-beneficial bacteria. For anti-inflammatory diets, nutritionists recommend consuming foods high in antioxidants. Food production for specific health or lifestyle requirements will be significantly impacted by enzymatic modifications, cautious glycation, and fermentation [136].

### 9.6. Regulatory Framework and Consumer Education

Having well-developed safety, function, and labeling requirements is crucial when new protein–carbohydrate conjugates emerge. By better educating people about the benefits and safety of novel chemicals, you may prepare the market for their adoption. Customers will view new technology more favorably if the sourcing process, food-processing methods, and health effects are made more transparent. Protein–carbohydrate interactions require new, accurate techniques, long-term advancements, and advances in technology as well as its practical use. The food business may create intelligent goods that are beneficial to both humans and the environment by utilizing bioinformatics, innovative processing techniques, artificial intelligence, and nutritional targeting.

## 10. Conclusions

In food science, protein–carbohydrate interactions are crucial because they affect the majority of food quality factors, including texture, taste, nutrition, and shelf life. Covalent (Maillard, controlled glycation) and non-covalent (hydrogen, electrostatic, hydrophobic) protein–carbohydrate interactions are now well-characterized at the mechanistic level, but their translation into commercial food products is uneven. Maillard-based flavor and color control is already commercially mature; controlled glycation for emulsifier replacement is still mostly laboratory-scale. Quantitative comparison across studies is limited by inconsistent assay protocols. The standardization of emulsifying activity index, gel strength, and AGE measurement methods is a near-term priority for the field. Plant-based dairy and meat analogs are the food matrices with the highest immediate commercial value for engineered protein–carbohydrate interactions, because they require simultaneous control of texture, mouthfeel, and protein quality. The principal safety question is the dietary AGE load from highly processed protein–carbohydrate conjugates. Regulatory frameworks for upper limits on AGE intake do not yet exist and should be prioritized. Computational and omics tools will accelerate formulation only if the food-science community publishes standardized, machine-readable interaction datasets. The current literature is rich in narrative reviews and poor in shared raw data.

Across the systems compared in Table 1, the same four dimensions (interaction type, food matrix, processing conditions, and functionality) consistently explain why comparable interaction chemistries yield different functional outcomes, confirming the value of an integrated rather than system-by-system analysis.

## Figures and Tables

**Table 1:** Representative protein–carbohydrate systems compared across the four framework dimensions.

Representative System	Interaction Type	Food Matrix	Processing Condition	Functional Outcome
Gluten-reducing sugar Maillard conjugate	Covalent (Maillard)	Wheat dough/bakery	High-temperature baking	Crust color, aroma, stiffer gluten network, gas retention
Whey/rice protein–amylose	Non-covalent (hydrogen bonding)	Starch-rich (noodles, bread)	Steaming, cooling/storage	Retrogradation control, softness retention, longer softness retention during storage
Protein–chitosan complex	Covalent + non-covalent	Coatings/films	pH/ionic-strength tuning, mild heating	Edible films, emulsion stabilizers, protective coatings
SLF protein–polysaccharide	Non-covalent (electrostatic + H-bond)	Low-fat ice cream/dairy	Cold processing, aeration	Melt resistance, creaminess, antioxidant capacity, lower cost
Okara protein–fiber	Non-covalent + bioconversion	Fermented plant matrix	Microbial fermentation	Improved gelation, antioxidant activity, digestibility
Pectin/cellulose–meat protein	Non-covalent (H-bond, van der Waals)	Processed/low-fat meat	Mixing, mild thermal set	Firmness, water retention, fat replacement

**Table 2:** Mechanistic and functional summary of protein–carbohydrate interaction types.

Interaction Type	Bond Character	Main Technological Effects	Sensory and Nutritional Impact	Representative Food Applications
Hydrogen bonding (non-covalent)	Weak, reversible	Gel stabilization, film cohesion, water retention	Smoother texture, controlled syneresis	Edible films, gluten-free dough, yogurt
Electrostatic (non-covalent)	Reversible, pH-sensitive	Coacervation, emulsion stabilization, microencapsulation	Improved colloidal stability of beverages	Milk–tea emulsions, dairy and plant–protein drinks
Hydrophobic (non-covalent)	Reversible	Interfacial activity, foam stabilization	Creaminess, fat-mimicking mouthfeel	Low-fat ice cream, meat analogs
Protein–oligosaccharide (non-covalent)	Reversible, branching-dependent	Foaming, viscosity control, prebiotic delivery	Improved gut-targeted nutrient release	Probiotic yogurts, fortified beverages
Protein–polysaccharide (non-covalent)	Reversible	Network formation, encapsulation, controlled release	Slowed digestion, lower glycemic index	Functional foods, nutraceutical delivery

**Table 3:** Quantitative effects of the protein–carbohydrate interactions reported in recent studies.

System	Effect Measured	Range	Reference
Rice starch/soy protein mixtures	Storage modulus G’ increase	Up to 30–40% control depending on ratio	[87]
Whey protein–pectin gels	Gel strength	Varies with 6.0 pH; max near 7.0 pH	[88]
Bovine serum albumin–starch conjugates (PEF-assisted glycation)	Emulsifying activity index improvement	Substantial increase over thermal-only control	[29]
Sweet lupine flour ice cream	Cost reduction vs. skim milk powder	~30%	[84]
Fermented okara	Antioxidant activity improvement	Using solvents (e.g., methanol or ethanol)	[89]

**Table 4:** Limitations of protein–carbohydrate interactions and mitigation strategies.

Limitation	Mechanism	Mitigation Strategy	Reference
AGE formation at high heat	Extended Maillard reaction with lysine	Lower-temperature processing; antioxidant addition; controlled water activity	[122]
Reduced lysine bioavailability	Early Maillard intermediate loss	Limit thermal load; use non-reducing sugars where possible	[123]
Batch-to-batch variability of plant ingredients	Variable DP and branching of pectin/inulin	Standardize raw-material specifications; pre-characterize molecular weight	[124]
Process control of PEF, ultrasound	High capital cost, fouling, denaturation risk	Pilot-scale process optimization; clean-in-place protocols; energy-input mapping	[125]
Consumer mistrust of engineered conjugates	Clean-label perception	Transparent labeling; fermentation-based glycation pathways	[126]

## Data Availability

No new data were created or analyzed in this study. Data sharing is not applicable to this article.
